# Cyclodextrin-Based Pickering Emulsion Significantly Increases 6-Gingerol Loading Through Two Different Mechanisms: Cyclodextrin Cavity and Pickering Core

**DOI:** 10.3390/foods14061066

**Published:** 2025-03-20

**Authors:** Xingran Kou, Dongdong Su, Jingzhi Zhang, Fei Pan, Jiamin Zhu, Qingran Meng, Qinfei Ke

**Affiliations:** 1 Key Laboratory of Textile Science & Technology, Ministry of Education, College of Textiles, Donghua University, Shanghai 201620, China; 2 Collaborative Innovation Center of Fragrance Flavour and Cosmetics, School of Perfume and Aroma Technology (Shanghai Research Institute of Fragrance & Flavour Industry), Shanghai Institute of Technology, Shanghai 201418, China; sudd0115@163.com (D.S.); mirrovo@163.com (J.Z.); zjmwyyxh@163.com (J.Z.); qmeng@sit.edu.cn (Q.M.); 3 State Key Laboratory of Resource Insects, Institute of Apicultural Research, Chinese Academy of Agricultural Sciences, Beijing 100093, China; yunitcon@yeah.net

**Keywords:** β-cyclodextrin, 6-gingerol, self-assembly, Pickering emulsion

## Abstract

We previously found that host–guest interactions can drive gingerols (Gs) and cyclodextrins (CDs) together to form inclusion complexes (G/CD), which can further construct amphiphilic microcrystals and resultant Pickering emulsions through self-assembly. In this follow-up study, we explored the detailed formation processes and mechanisms of the 6-G/β-CD inclusion complex and the resultant Pickering emulsion. The influence of the 6-G/β-CD molar ratio on the structure, morphology, and loading capacity of the inclusion complex and resultant Pickering emulsion were investigated. The results show that the cyclodextrin-based Pickering emulsion can load 6-G in two places; one place is the cyclodextrin cavity, whose loading capacity is up to 9.28%, while the other one is the Pickering core, with its highest loading capacity at 32.31% when the 6-G/β-CD molar ratio is 5:1. In the above case, the 6-G/β-CD inclusion complex was found to form a unit cell with a 1:2 molar ratio and then self-assemble into amphiphilic microcrystals through cage-type arrangement structures at the oil–water interface, mainly driven by van der Waals forces and hydrogen bonds. This study is helpful in the design and preparation of CD-based high-loading carriers for bioactive compound delivery.

## 1. Introduction

6-gingerol (6-G) is the main active ingredient in ginger, and has health benefits including anti-inflammatory, antiviral, anti-tumor, antioxidant, and antiemetic properties [1]. However, its low water solubility limits its application in functional foods, cosmetics, medicine, and other fields. Fortunately, diverse strategies have been explored to enhance the delivery of 6-G, such as nanovesicle-based 6-G/hydroxypropyl-β-cyclodextrins (HP-β-CD) inclusion complexes and non-ionic surfactants [2], as well as microemulsions formulated with ethyl oleate, polyoxyethylene castor oil EL35, or 1,2-propylene glycol [3]. Some inclusion complexes have been developed to utilise protein-based biowalls such as bovine serum protein, among other materials [4]. Lipid carriers composed of monostearoyl glycerides, decanoyl/octanoyl glycerides, and mixed surfactants have also been studied for this purpose [5]. However, the current drug loading capacity for 6-G remains limited, typically ranging between 1.17% and 4.64% [5,6,7].

CD-based Pickering emulsions have emerged as a versatile emulsification system in recent drug delivery research [8]. CDs represent cyclic oligosaccharides characterised by structures comprising six (α-CD), seven (β-CD), eight (γ-CD), or more glucopyranose units linked by α-1,4 glucoside bonds [9]. Their distinctive hollow ring structure enables the encapsulation of various molecules within their interiors, while the hydrophilic nature of their hydroxyl groups ensures stability in aqueous environments. In Pickering emulsions, solid particles such as CDs and guest/CD inclusion complexes adsorb at the oil–water interface, forming either a single- or multi-layer structure. This effectively immobilises the interface between the oil and water phases, imparting exceptional stability and rheological properties to the emulsion [10,11,12]. These surfactant-free formulations exhibit improved physical stability, tolerance, and environmental compatibility compared to traditional surfactant-based emulsions [10]. In drug-carrier selection research, natural materials such as proteins and polysaccharides hold great potential for advancing Pickering emulsion formulations, owing to their widespread availability, cost-effectiveness, and eco-friendliness [13]. These natural materials possess intricate and diverse solid particle morphologies and compositions, coupled with commendable biocompatibility and regulatability [14].

Our investigation revealed that amphiphilic microcrystals, which formed through the inclusion complexes of ginger oleoresin/β-CD, promoted the formation of Pickering emulsions [15]. However, owing to the intricate composition of ginger oleoresin [16,17], the interaction mechanism between the ginger oleoresin components and β-CD is poorly understood. Although one prior investigation has scrutinised the preparation and characterisation of the 6-G/β-CD inclusion complex [18], the potential of this complex to engender Pickering emulsions remains unclear.

This study systematically investigated the formation processes and underlying mechanisms of the 6-G/β-CD inclusion complex and its role in stabilising Pickering emulsions. The characterisation of the system was conducted using optical microscopy, phase solubility analysis, thermogravimetric analysis, and powder X-ray diffraction (XRD). Our study demonstrated that 6-G and β-CD form stable inclusion complexes via host–guest interactions, enhancing 6-G’s aqueous solubility. The 6-G loading occurs through β-CD cavity encapsulation or physical adsorption in the Pickering emulsion core, improving dispersibility. The self-assembled amphiphilic microcrystals stabilise the oil–water interface, promoting emulsion formation. Molecular dynamics (MD) simulations further elucidated the self-assembly process and interaction mechanisms. These findings highlight the potential of this system to enhance the aqueous dispersion of 6-G and provide valuable insights for the development of bioactive compound delivery platforms.

## 2. Materials and Methods

### 2.1. Materials and Chemicals

**Figure 1:**
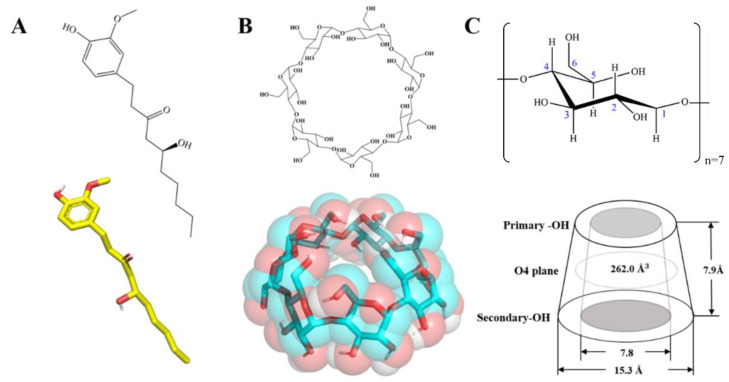
Molecular structures of 6-G and β-CD. (**A**) 6-G; (**B**) β-CD; (**C**) Schematic representation of glucose unit and cone-columnar structure of β-CD.

β-CD (purity ≥ 98%) was procured from Adamas Reagents. High-purity ginger oleoresin, with a high-performance liquid chromatography (HPLC) ≥ 37.01% of 6-G concentration, was obtained from Lihe Flavor (Qingdao) Food Industry. The 6-G (Figure 1) utilised in the sample preparation underwent extraction from the highly purified gingerol through multiple chromatography steps (HPLC ≥ 93.87%; details in Supplementary Data S1). Table S1 lists the purity detection of different 6-G samples, while Figure S1 shows their HPLC detection spectra. A 6-G standard (No. CHB-J-024; HPLC ≥ 98%) was obtained from Chengdu Cloma Biotechnology. Absolute ethanol (purity ≥ 99.7%) was supplied by Shanghai Kolins Reagent. In-house laboratory-distilled water was employed for all experiments.

### 2.2. Sample Preparation

The 6-G/β-CD inclusion complexes were prepared as described by Nuchuchua et al. [19], with slight modifications. Initially, a saturated aqueous solution of β-CD (5.27 g/mL) was prepared at 50 °C. Each specified quantity of 6-G (the 6-G/β-CD molar ratios were set to 0.0:1, 0.1:1, 0.25:1, 0.5:1, 0.5:1, 1.0:1, 2.0:1, and 5.0:1, respectively) was combined with 20 mL of the prepared β-CD aqueous solution and stirred at 50 °C and 800 rpm for 24 h, left to cool to 25 °C, and stored in darkness until utilisation. Physical mixtures of 6-G and β-CD were obtained by lyophilising the two in a ratio of 0.5:1 and stirring with a stir bar.

In order to obtain purer inclusion complexes, we selected three treatments to process the samples (Supplementary Data S2). Figure S2 demonstrates the UV absorption intensities of the 6-G/β-CD inclusion complexes obtained by the three treatments. Finally, we used a vacuum suction filter to isolate the purified 6-G/β-CD inclusion complex powder. Initially, the samples were applied onto a 50 mm × 0.45 μm organic phase filter membrane. The excess 6-G was eliminated through four cycles of 30 mL of absolute ethanol, while the residual β-CD was washed using four cycles of 30 mL of distilled water to determine the appropriate volume of irrigating fluid. The resulting sample of the pure 6-G/β-CD inclusion complex was retained on a filter membrane and freeze-dried under a vacuum at −50 °C for 12 h to yield a white 6-G/β-CD powder sample, which was stored in an airtight bottle in a dry environment.

### 2.3. Microscopic Observation

We acquired optical images using Olympus DP74 test software (v4.1) integrated with an Olympus VR BX 53 microscope (Olympus, Tokyo, Japan).

The surface topography of the samples was captured employing a Gemini SEM 300 scanning electron microscope (SEM, ZEISS, Oberkochen, Germany) operating at an acceleration voltage of 3 kV for topographical imaging and 15 kV for energy spectrum mapping. An SE2 secondary electron detector was utilised as the primary detection mechanism.

### 2.4. Determination of 6-G Loading Capacity and 6G/β-CD Molar Ratio

To prepare a 6-G standard curve, we dissolved 0.031 g of 6-G in absolute ethanol within a 100 mL volumetric flask to generate a stock solution. We prepared 10 distinct solutions by accurately diluting the stock solution in 10 mL volumetric flasks, resulting in 6-G concentrations of 0.31–0.031 mg/mL. Employing absolute ethanol as the blank control, the absorbance values of each solution were measured at a wavelength of 190–400 nm. The acquired data were graphically represented, with the absorbance value at the maximum absorption wavelength plotted on the ordinate and the corresponding 6-G concentration on the abscissa.

The leached and non-leached 6-G/β-CD powder sample (0.015 g) was dissolved in 10 mL of absolute ethanol and stirred at 60 °C and 800 rpm for 20 min to ensure complete dissolution. The resulting solution was filtered through a 0.22 μm nylon filter membrane and subjected to 10-fold dilution with absolute ethanol and sonication for 20 min to create the test solution [4]. The absorbance at the maximum wavelength of 6-G was measured using a UV–visible spectrophotometer (Hitachi, U-3900, Tokyo, Japan), with absolute ethanol employed as a blank control. The mass of 6-G in the sample was determined through calibration with the standard curve, facilitating the calculation of the 6-G loading capacity (LC%) using Equation (1):

(1) $$LC\%=\frac{m_{1}}{m_{0}}\times 100\%.$$

Here, $m_{1}$ represents the mass of 6-G in the sample, and $m_{0}$ represents the total mass of the sample.

Equation (2) was used to determine the molar ratio of 6-G to β-CD within the inclusion complex. This methodology ensures a systematic and rigorous approach to quantifying the 6-G loading and evaluating the molar ratio in the inclusion complex.

(2) $$Molar\;ratio\;of\;6G\;to\;\beta -CD\;=\;\frac{m_{1}/294}{(m_{0}-m_{1})/1134}$$

### 2.5. Phase Solubility Measurement

At 25 °C, a 0.010 mol/L aqueous solution of β-CD was prepared by dissolving 1.134 g of β-CD in 100 mL of distilled water. This solution was subsequently diluted at concentration gradients of 0.010, 0.008, 0.006, 0.004, 0.002, and 0 mol/L. In a container containing an excess of 6-G (0.020 mol/L, 0.0588 g), 10 mL of the aqueous solutions with varying concentrations of β-CD were sequentially added. The sample preparation was finalised by magnetic stirring at 800 rpm for 18 h at 50 °C. Prior to the UV–visible spectrophotometer analysis, the samples were filtered through a 0.45 μm hydrophilic polyvinylidene chloride filter membrane. The filtrate was diluted tenfold with absolute ethanol and sonicated for 20 min to yield the sample test solution.

Absolute ethanol served as the blank control for assessing the absorbance of 6-G at its maximum absorption wavelength. A phase solubility map was constructed by plotting the solubility of 6-G in the solvent (mM/L) against the concentration of β-CD (mM/L), which employed the 6-G standard curve calculations. If the resulting phase solubility diagram conformed to the $A_{L}$ type, signifying a linear increase in 6-G solubility with β-CD concentration, the inclusion constant (K) was computed using Equation (3). In other cases, linear regression was applied to calculate the inclusion constants under varying conditions according to Equation (3):

(3) $$K=\frac{10^{3}k}{d(1-k)}$$

Here, K is the inclusion constant (L/mol), k is the slope of the linear regression equation in the phase solubility diagram, and d is the intercept of the linear regression equation in the phase solubility plot (mmol/L). This systematic approach facilitates a comprehensive analysis of the inclusion phenomena and their dependence on the β-CD concentration [20].

### 2.6. Thermogravimetric Analysis

Thermogravimetry (TG) and its first derivative (DTG) were measured using a TGA/DSC 3+ calorimeter and thermogravimetric analyser (Mettler Toledo, Zurich, Switzerland) and STARe thermal analysis software (v14.00). Equal amounts (8.5 mg) of the freeze-dried β-CD, 6-G/β-CD inclusion complex, and physical mixture powders were weighed, and 30 mg of 6-G oily liquid was weighed. The samples were heated from 30 °C to 600 °C at a heat rate of 10 °C/min in a dynamic high-purity nitrogen atmosphere of 50 mL/min.

### 2.7. Powder XRD

XRD analysis was conducted using a D8-VENTURE diffractometer (Bruker, Karlsruhe, Germany) to generate the diffraction patterns for β-CD, the physical mixtures of 6-G and β-CD, and the 6-G/β-CD inclusion complexes. The data acquisition employed CuKa radiation (λ = 1.54178 Å) with a CMOS-based Photon 100 detector. The XRD was operated at 40 kV and 40 mA, a scanning speed of 2°/min, and an angular range of 5–50°. Subsequently, the collected data were refined through a Rietveld full spectrum fitting using Jade 9.0. A comparative analysis was performed against the PDF2016 standard atlas database. Finally, the average particle size was determined, utilising the Debye–Scherer formula as follows [21]:

(4) $$D=\frac{k\lambda }{\beta_{0}\cos\theta },$$

where D is the particle size, k is the shape factor (0.89), λ represents the wavelength of the X-ray, $\beta_{0}$ is the FWHM of the diffraction peak, and θ is the Bragg diffraction angle.

### 2.8. MD Simulation Setup

#### 2.8.1. Model Construction

According to the current experimental evidence, 6-G and β-CD most commonly exist in a 1:2 molar ratio structure. β-CD dimers often have the most stable head-to-head and tail-to-tail binding modes [22,23,24]. Therefore, when exploring the structure of the inclusion complex between 6-G and β-CD, we focused specifically on the inclusion complex consisting of 6-G and a head-to-head β-CD dimer at a 1:2 molar ratio. The molecular structure of the head-to-head β-CD dimer was downloaded from the Cambridge Crystallography Data Centre (CCDC ID: 1955632), and the crystal structure was an inclusion complex of geranyl alcohol/β-CD [25]. PyMol was used to remove the redundant structure in the downloaded file [26], and an MMFF94s force field in Avogadro software (v1.2.0) was used to optimise the structural mechanics of each molecule in terms of the geometric and energy parameters to minimise the overall potential energy of the structure [27]. The Automatic Topology Generator and Repository version 3.0 database [28] was used to obtain the 6-G molecular file optimised based on the Hessian calculation and the DFT theoretical level (B3LYP/6-31G *) under all-atom approximation, corresponding to the ATB number 225341 [29]. Docking via Autodock Vina software (v1.1.2) showed that the β-CD dimer as a whole was combined with 6-G, and the best docking conformation was used in the subsequent simulation [30].

For the emulsion model, first, 600 6-G molecules were inserted into a 15 × 15 × 10 nm^3^ box as the oil phase system. After energy minimisation, NVP, NPT, and running for 10 ns, the box system size became 7.52181 × 7.52181 × 5.0436 nm^3^. Then, the box was enlarged along the *z*-axis to construct a 7.52181 × 7.52181 × 15.0436 nm^3^ system. The amplified space was solvated so that the SOL water molecules filled the system; the water molecular layer was finally filled with 6-G/β-CD inclusion complexes.

#### 2.8.2. Simulation Parameters

MD simulations spanning 180 ns were executed using the GROMACS 19.5 program, employing the charmm36-jul2020 force field and explicit solvation [31]. The CGenFF Web server (http://mackerell.umaryland.edu/cgenff_dwld.php, accessed on 16 March 2025) facilitated the generation of the simulation files, which require charmm36 force field parameters. The simulation adopted periodic boundary conditions, ensuring a minimum distance of 10 Å between any atom and the box edge [32]. The protocol commenced with the energy minimisation, using the steepest descent method. Subsequently, the canonical ensemble (NVT, 0.1 ns, 300 K) and the isothermal–isobaric ensemble (NPT, 0.1 ns, 1 bar) pre-balancing procedures were implemented [33,34]. The time step for the energy minimisation, NVT, NPT, and MD stages was set at 2 fs, with the other parameters set as previously described [35].

#### 2.8.3. Visualisation Method of Simulation Process

The visualisations and graphical representations of the extracted conformations were achieved using PyMol (v1.8.6) [26] and VMD (v1.9.3) [36] software, respectively.

#### 2.8.4. Weak Interaction Analysis

For an in-depth exploration of weak intramolecular and intermolecular interactions, Multiwfn was employed as an independent gradient model (IGM) [37]. Additionally, VMD was utilised to generate coloured isosurface maps [38,39].

## 3. Results and Discussion

### 3.1. Sample Morphology

**Figure 2:**
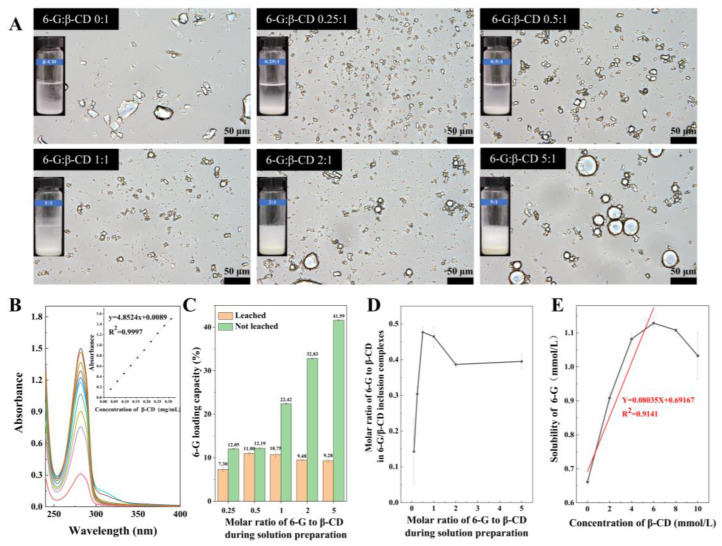
Microscopic investigation of 6-G/β-CD. (**A**) Light microscopy. (**B**) 6-G UV–visible spectrophotometer scan and standard curve. (**C**) Loading capacity of 6-G. (**D**) Relationship between loading molar ratio of 6-G to β-CD during solution preparation and their molar ratio in resulting 6-G/β-CD solid inclusion complex. (**E**) Phase solubility plots and linear fits of 6-G at different β-CD concentrations.

Optical microscopy facilitated the rapid assessment of the sample morphology in the liquid state (Figure 2A). β-CD exhibited a diverse block structure with dimensions ranging from 2 to 50 μm [40]. Notably, the samples prepared with a 0.25:1 molar ratio of 6-G to β-CD generated small and uniform crystals (~10 μm). Intriguingly, many regular sheet rhomboid structures were observed with the 0.5:1 molar ratio treatment. Conversely, the samples prepared at molar ratios of 1:1, 2:1, and 5:1 displayed random globular-like droplets, surrounded by numerous sheet or stick-shaped crystal fragments—a characteristic manifestation of Pickering emulsions [41].

### 3.2. Loading Capacity of 6-G

#### 3.2.1. 6-G Standard Curve

The UV–vis spectrophotometer analysis (in the wavelength of 190–400 nm) revealed the maximum absorption wavelength of the ethanol-dissolved 6-G at 282 nm. The 6-G standard curve (Figure 2B) illustrates the linear relationship between the 6-G concentration and absorbance value at 0.031–0.310 mg/mL. The linear regression equation obtained through linear fitting demonstrated a robust linear relationship (Y = 4.8524X + 0.0089, $R^{2}$ = 0.9997) and enabled the calculation of the 6-G concentration within the absorbance range of 0.161–1.5.

#### 3.2.2. Loading Capacity of 6-G in Pickering Emulsion

In our investigation, distinctive spaces for drug accommodation were identified within the amphiphilic solid particles generated by 6-G and β-CD, specifically the inclusion complex microcrystals (Figure 2C). The 6-G/β-CD inclusion complex, obtained via leaching treatment, exhibited a minor variation in 6-G loading (7.30–11.00%), demonstrating the efficacy of leaching for determining the inclusion ratio of 6-G within β-CD inclusion complexes.

In the sample without leaching treatment, the 6-G loading amount during preparation was positively correlated with the initial addition amount [11,42]. However, the increase in loading capacity was significantly smaller than the corresponding increase in addition. Notably, the 6-G loading of the Pickering emulsion was significantly improved to 41.59% from 9.28% at a 6-G to β-CD molar ratio of 5:1. This finding highlights the dual role of the 6-G/β-CD inclusion complex in improving the loading capacity of Pickering emulsions, as evidenced by the β-CD cavity load of 9.28% and the Pickering core load of 32.31%. This finding validates the applicability of solid CD-based inclusion complexes in Pickering emulsion formation.

#### 3.2.3. Molar Ratio in Inclusion Complexes

Figure 2D presents the correlation between the molar ratios of 6-G to β-CD during solution preparation and the resultant molar ratios in the solid 6-G/β-CD inclusion complexes. Between 0:1 and 0.5:1 of the molar ratio of 6-G to β-CD during solution preparation, the addition of 6-G increased its molar proportion in the corresponding inclusion complexes. Upon surpassing a critical threshold, the molar ratio of the inclusion complex stabilised (fluctuating around 0.4–0.5), with the highest recorded value being 0.4766 ± 0.0145. Consequently, the stoichiometry of the 6-G/β-CD inclusion complex was inferred to be 1:2 [19]. Remarkably, when the 6-G to β-CD molar ratio during solution preparation exceeded 2:1, the molar ratio within the inclusion complex decreased with the increasing 6-G concentration in the solution. The surplus 6-G in the solution tended to self-aggregate, forming a Pickering core region characterised by higher hydrophobicity than the β-CD cavity; this led to the spontaneous detachment of 6-G from the β-CD cavity within the 6-G/β-CD inclusion complex. Simultaneously, the 6-G oil droplets in the Pickering emulsion exhibited greater susceptibility to removal during the absolute ethanol leaching than the 6-G/β-CD inclusion complex.

### 3.3. Phase Solubility Analysis

Water solubility is a key physicochemical property determining the bioavailability of organic small-molecule drugs [43]. In medicinal chemistry, drug water solubility is often improved by generating a CD-based hydrophilic shell [44,45]. The CD inclusion process is complex and directly affects the shape, geometric size, and molecular configuration of the encapsulated molecules. The solubilisation of 6-G by β-CD at different concentrations was compared, and Figure 2E shows that the phase solubility diagram of 6-G in the β-CD solution conforms to type $A_{N}$. Similarly, in the phase solubility of α-, β-, γ-, and hydroxypropyl-β-CD in the inclusion of anisette essential oil, the phase solubility diagram conformed to the $A_{L}$ type, while the phase solubility diagram in the β-CD solution conformed to the $A_{N}$ type, which may be related to the enhanced formation of supramolecular polymers from β-CD and its inclusion complexes.

Through the linear fitting method, we fit the data for β-CD concentrations of 0–6 mmol/L [24] and obtained the linear relationship Y = 0.08035X + 0.69167 ($R^{2}$ = 0.9141). According to Equation (3), the inclusion constant K at this time was 126.3177 L/mol. Notably, the formation of the 6-G/β-CD inclusion complexes promoted the dissolution of 6-G. At 6 mmol/L β-CD, the solubility of 6-G increased 1.7067-fold, which was higher than the 1.50-fold increase caused by bovine serum albumin [4]. These data suggest that β-CD is a useful solubilising adjuvant in 6-G drug delivery systems.

### 3.4. Validation of 6-G/β-CD Inclusion Complex Formation

**Figure 3:**
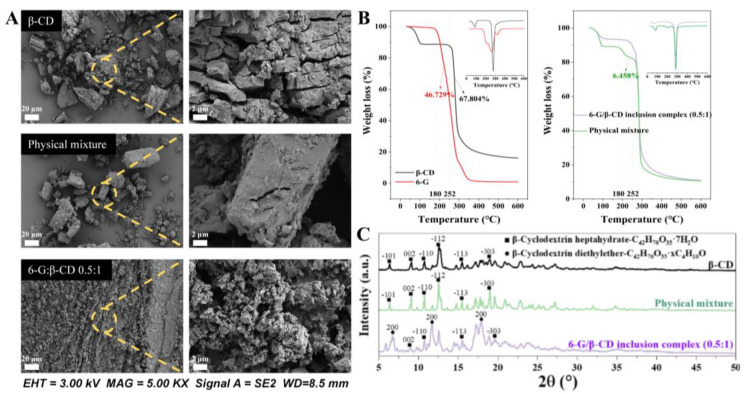
Validation of 6-G/β-cyclodextrin inclusion complex formation. (**A**) SEM diagram; (**B**) TG and DTG curves; (**C**) XRD patterns.

SEM was utilised to examine the morphological characteristics of β-CD, the physical mixtures of 6-G and β-CD, and the inclusion complex formed by 6-G/β-CD. The physical mixtures displayed irregular crystal lumps, with the crystal particles clustered on an uneven surface, presenting an overall loose state (Figure 3A) consistent with previous observations [18,46]. In contrast to β-CD in an aqueous solution, the β-CD powder exhibited a distinct loose fracture state attributed to the physical interactions in the absence of water [47].

Distinct from earlier observations of the 6-G/β-CD binary inclusion complex, characterised as club-like [18], the 6-G/β-CD inclusion complex prepared at a molar ratio of 0.5:1 featured smaller and relatively uniformly sized particles. However, the inter-particle aggregation was comparatively weak, and the particles exhibited a more regular sheet diamond shape. These differences may be attributed to variations in the quantity of β-CD added and the temperature during preparation and crystal formation.

TG and DTG constitute standard methodologies for investigating mass variations during heating. The determination of inclusion complex formation typically involves a comparative analysis of the thermogravimetric curves derived from the physical mixtures and inclusion complexes [48]. Figure 3B depicts the TG curves representing β-CD, the physical mixtures of 6-G and β-CD, and the inclusion complexes formed by 6-G/β-CD. Notably, we observed a distinct contrast in thermal decomposition behaviour between the pure 6-G and β-CD substances (left side of Figure 3B). At 180–400 °C, the respective weight losses for 6-G and β-CD were 96.73% and 69.042%, respectively. At 180–252 °C, 6-G lost 46.729% of the initial weight, while β-CD lost only 1.238%. The weight loss profiles of 6-G and β-CD partially overlapped at 252–400 °C, at which point the composition ratio cannot be effectively discerned for the inclusion complexes [49].

We compared the thermal profiles of the physical mixtures with the 6-G/β-CD inclusion complexes (right side of Figure 3B) and observed the complete absence of a 6-G peak in the inclusion complexes at 180–252 °C. The physical mixtures demonstrated more rapid and severe weight loss than the 6-G/β-CD inclusion complexes across the three principal weight loss stages: 30–110 °C, 180–252 °C, and 252–400 °C [50]. These findings validate the effective encapsulation of 6-G within the β-CD cavity and the successful formation of the 6-G/β-CD inclusion complex.

### 3.5. 6-G/β-CD Inclusion Complex Structure

We performed a comparative analysis of the XRD spectra between the CDs and inclusion complexes to assess the integration of guest molecules within the CD cavity and the crystal properties within these complexes [21,51]. Figure 3C illustrates the diffraction patterns within the 5–50° range. Notably, the β-CD spectrum had six absorption peaks aligning with the β-CD·7H_2_O phase standard card [52], representing the characteristic β-CD peaks. The physical mixtures of 6-G and β-CD showed a strong and sharp diffraction peak shape. The altered diffraction peak of the 6-G/β-CD inclusion complex suggested a significant modification in the crystal structure of 6-G following its successful encapsulation by β-CD. Specifically, at 12.590°, the disappearance of the most pronounced characteristic diffraction peak of β-CD implied a perturbation in its original crystal structure during encapsulation. Conversely, new robust characteristic peaks emerged at 11.63°, 17.21°, and 17.79°, indicative of the novel crystalline phases and affirming the establishment of the 6-G/β-CD inclusion complexes [53]. These three peaks corresponded to the distinctive “cage-type” structure of the 6-G/β-CD inclusion complex, consistent with previous findings [54].

The average particle sizes of β-CD, the physical mixtures of 6-G and β-CD, and the 6-G/β-CD inclusion complexes were 379 Å, 816 Å, and 141 Å, respectively. This finding revealed that the encapsulation of 6-G within the β-CD cavity disrupts the lattice periodicity of β-CD, thereby reducing the average particle size [55]. This phenomenon was consistent with the XRD and microscopy observations. Simultaneously, the decrease in particle size contributed to the broadening of the XRD peak [56]. Notably, the 6-G/β-CD inclusion complex exhibited additional diffusion peaks and reduced crystallinity, indicating an encapsulation-induced amorphous state. Despite the reduced crystallinity, this amorphous state is associated with enhanced 6-G bioavailability [57]. In contrast, recrystallised cores formed around the extruding regions in the physical mixtures (prepared with mechanical agitation), which can accelerate the rate of annexation and sustain the core-to-core growth, resulting in larger particles [58].

### 3.6. MD Simulation

#### 3.6.1. Visualisation of Simulation Process

**Figure 4:**
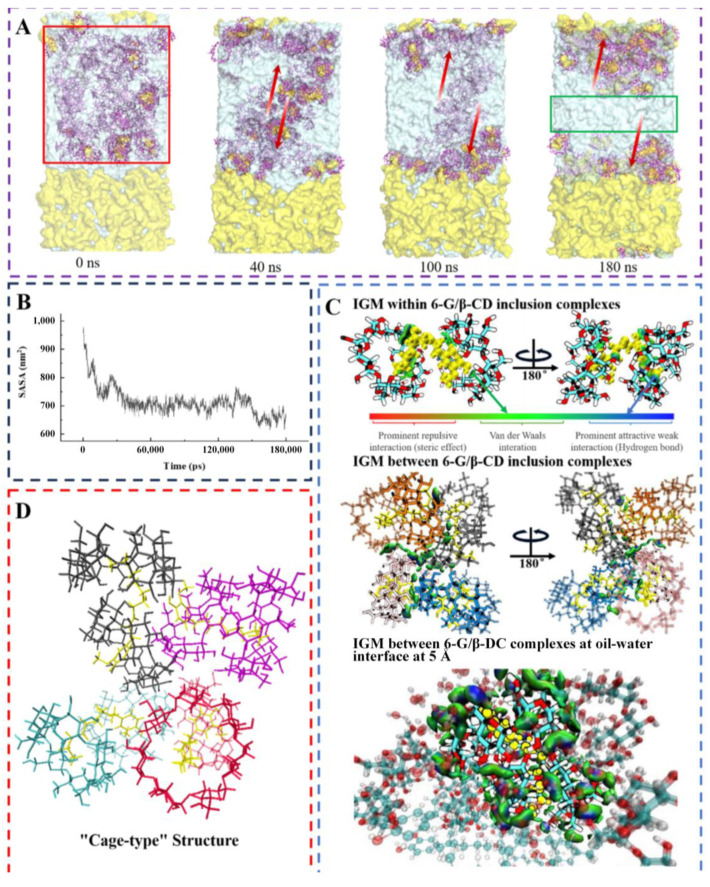
MD simulation analysis. (**A**) Visualisation of simulation process; (**B**) Solvent-accessible surface area of 6-G/β-CD inclusion complexes; (**C**) IGM within, between, and at oil–water interface within 5 A of 6-G/β-CD inclusion complexes; (**D**) “Cage-type” structure formed between 6-G/β-CD inclusion complexes.

Figure 4A depicts the dynamic behaviour of the 6-G/β-CD inclusion complex at the water–oil interface. At 0 ns, the 6-G/β-CD inclusion complex occurred within the aqueous phase. Over time, its amphiphilicity promoted its spontaneous migration towards the oil–water interface. We visualised the solvent-accessible surface area of the 6-G/β-CD inclusion complex (Figure 4B) and found its interaction with water molecules gradually decreased as it self-assembled toward the oil–water interface. This finding confirms the experimental Pickering emulsion formation and describes the amphiphilic nature of the 6-G/β-CD inclusion complex.

#### 3.6.2. Interaction Analysis

We performed an IGM analysis to elucidate the intramolecular and intermolecular interactions of the 6-G/β-CD inclusion complex, as well as the intermolecular interactions of the 6-G/β-CD inclusion complex at the oil–water interface at 5 Å (Figure 4C). The self-assembly of the 6-G/β-CD inclusion complex at the oil–water interface was primarily governed by van der Waals interactions and hydrogen bonding [59]. The prominent green area on the isopotential surface signifies that the van der Waals interactions are pivotal in maintaining the stability of the 6-G/β-CD inclusion complex and its aggregates. The blue regions denote hydrogen bonding interactions. The hydrophilic edge of β-CD in the 6-G/β-CD inclusion complex facilitates the conversion of the surrounding free water into bound water through hydrogen bonding [60]. This not only promotes the water solubility of the inclusion complexes, but also sterically stabilises the entire system by creating supramolecular self-assemblies along with the bound water [61].

The striking agreement between these model calculations and experimental phenomena demonstrates the reliability of our theoretical model. Through in-depth study of the packing structure of the 6-G/β-CD inclusion complex at the oil–water interface, we found a “cage-type” structure of misalignment between the complexes, which is particularly common in β-CD and its inclusion complex, especially in the monoclinic space group ${P2}_{1}$ [62]. Notably, this “cage-type” structure was formed by multiple inclusion complexes with two β-CDs and a 6-G. In the “cage-type” structure, the cavity edge of the β-CD cavity is tightly surrounded by the side or cavity edge of its neighboring molecule, enhancing the intermolecular interaction (Figure 4D). This close stacking not only improves the stability of the inclusion complex at the oil–water interface, but also facilitates the formation and maintenance of the solid layer, thereby significantly enhancing the stability of the emulsion [63].

## 4. Conclusions

In this study, a microcrystalline inclusion complex of 6-G and β-CD was successfully constructed, which effectively promoted Pickering emulsion formation and greatly improved the dispersion of 6-G in water. The loading analysis showed that the Pickering emulsion formed by the 6-G/β-CD inclusion complex exhibited a dual drug loading mechanism: the β-CD cavity encapsulated 9.28% of the drug, while the core part of the emulsion physically adsorbed 32.31% of the drug. This phenomenon not only greatly improves the dispersion and stability of the drugs in water, but also provides the possibility of controlled drug release. Through the phase solubility method and MD simulations, the 6-G/β-CD inclusion complex was found to form a unit cell with a 1:2 stoichiometry ratio, and then self-assemble into a “cage-type” structure at the oil–water interface, driven by van der Waals forces and hydrogen bonds. However, despite our attempts to use XRD techniques to resolve the structure, more precise details about the “cage-type” structure have not been obtained in this study due to a variety of factors, such as sample preparation issues, XRD experimental condition limitations, and difficulties in data processing and analysis. In addition, due to the extreme sensitivity of the 6-G/β-CD inclusion complex to electron beams, the diffraction spot of the structure could not be imaged by transmission electron microscopy. Therefore, the “cage-type” structure conclusion obtained from the MD simulations still needs to be further verified. In future studies, researchers should use more new methods to overcome these problems. 

## Data Availability

The original contributions presented in the study are included in the article/Supplementary Material, further inquiries can be directed to the corresponding author.

## Supplementary Materials

The following supporting information can be downloaded at: https://www.mdpi.com/article/10.3390/foods14061066/s1.
