# Supplementary material for: Cyclodextrin-Based Pickering Emulsion Significantly Increases 6-Gingerol Loading Through Two Different Mechanisms: Cyclodextrin Cavity and Pickering Core
Source: Foods. 2025 Mar 20;14(6):1066. doi: 10.3390/foods14061066 (PMC11942410; doi:10.3390/foods14061066)
Supplement: Supplementary file 1 [file foods-14-01066-s001.zip › foods-3519254-supplementary.pdf]

Cyclodextrin-Based Pickering Emulsion Significantly Increases 6-Gingerol Loading Through Two Different Mechanisms: Cyclodextrin Cavity and Pickering Core

Xingran Kou [1,2,*], Dongdong Su [2], Jingzhi Zhang [2], Fei Pan [3] , Jiamin Zhu [2], Qingran Meng [2] and Qinfei Ke [1,2,*]

[1] Key Laboratory of Textile Science & Technology, Ministry of Education, College of Textiles, Donghua University, Shanghai 201620, China

[2] Collaborative Innovation Center of Fragrance Flavour and Cosmetics, School of Perfume and Aroma Technology (Shanghai Research Institute of Fragrance & Flavour Industry), Shanghai Institute of Technology, Shanghai 201418, China; sudd0115@163.com (D.S.); mirrovo@163.com (J.Z.); zjmwyyxh@163.com (J.Z.); qmeng@sit.edu.cn (Q.M.)

[3] State Key Laboratory of Resource Insects, Institute of Apicultural Research, Chinese Academy of Agricultural Sciences, Beijing 100093, China; yunitcon@yeah.net

***** Correspondence: kouxr@sit.edu.cn (X.K.); kqf@sit.edu.cn (Q.K.)

Supplementary Data S1:

6-G extracted from highly purified gingerol by multiple chromatography and detected by external standard method using HPLC

Liquid chromatography conditions: Qualitative and quantitative analysis of gingerols was performed using a Shimadzu Agilent1100 high performance liquid chromatograph (HPLC), and all samples were filtered through 0.22 μm microporous membranes before injection. The volume of each injection was 20 uL, the column temperature was 25℃, the flow rate was 1mL/min, the chromatographic column was HT-330-C18 column (5 μm, 4.6 mm×250 mm), the mobile phase was acetonitrile and water, and the volume ratio of acetonitrile was 35%-70% during 0-30min. Under these conditions, 6-G can be separated from other substances in the reagent. Retention time and peak area were recorded, and 6-G content was detected by external standard method.

Results: After three repeated experiments, the ratio of 6-G absorption peak area to the total absorption peak area was finally increased from 38.48% ± 1.83% to 94.99% ± 1.12% (Table S1).

Table S1. Sample purity detection

Sample	6-G absorption peak area to the total absorption peak area
6-G standard	99.09% ± 0.29%
Highly purified gingerol	38.48% ± 1.83%
6-G phenol extraction results	94.99% ± 1.12%

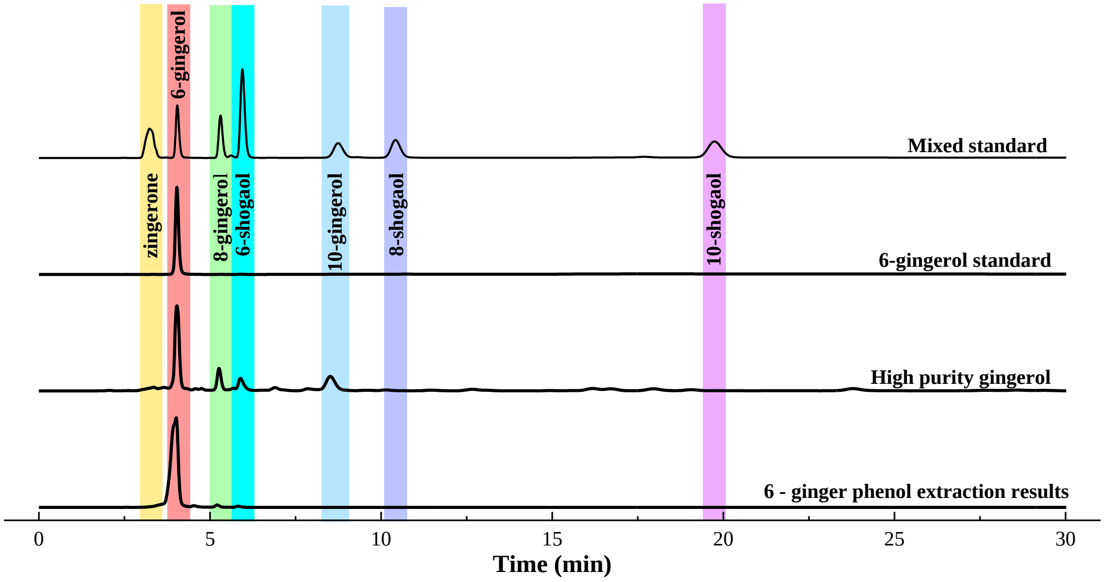

Figure S1. HPLC detection pattern

Supplementary Data S2:

Exploration of treatments to obtain pure 6-G/β-CD inclusion complexes

Ensuring the purity of the 6-G/β-CD inclusion complex is essential for accurate calculation of drug loading. The presence of unbound 6-G in the 6-G/β-CD inclusion complex may lead to an overestimation of the load, whereas overremoval of excess β-CD also may lead to an underestimation of the load. Therefore, the exploration of treatments to obtain pure 6-G/β-CD inclusion complexes is necessary. Existing methods for the treatment of CDs inclusion complexes usually involve membrane filtration or leaching steps, but the experimental details of these methods are vague, leading to doubts about the comparability and conclusions between different studies. Specifically, membrane filtration may leave unbound drug or CDs molecules, while leaching process may result in inconsistent results due to the lack of a clear elution amount. This ambiguity may lead to uncertainty in the purity of inclusion complexes, which in turn affects the accurate calculation of drug loading. To address this problem, we carried out three different treatments on the existing 6-G/β-CD inclusion complex preparation solutions to obtain pure 6-G/β-CD inclusion complex. The purified complexes were then used to determine the absorbance of 6-G at its maximum absorption wavelength using a UV-Vis spectrophotometer. This approach aimed to enhance the comparability and reliability of conclusions across different studies.

Treatment 1: A 0.45 μm hydrophilic filter membrane was used for filtration. First, the sample solution was allowed to stand for 3 h in order to isolate the clear layer, which is a pure 6-G/β-CD inclusion complex solution capable of dissolving in water. Then, the filtered sample was taken and diluted 10-fold with absolute ethanol, followed by 20 min of sonication. Finally, after centrifugation at 4000 rpm for 10 min, the supernatant was obtained as the sample to be tested.

Treatment 2: The sample was evenly spread on an organic phase filter membrane with a size of 50 mm*0.45 um, and first leached twice with absolute ethanol (30 mL/time), aiming to remove excess 6-G. This was followed by two additional leaches (30 mL each) with distilled water to remove excess β-CD. In this way, a sample of pure 6-G/β-CD inclusion complex on the filter membrane was obtained. The sample was dried by vacuum drying for 12 h at -50 ° C to obtain a white powder sample, which was placed in a drying dish to prevent moisture absorption.

Treatment 3: To further explore the effect of leaching times on obtaining reliable and pure 6-G/β-CD inclusion complex, on the basis of maintaining the same leaching

operation process as that of treatment 2, the number of leaching in absolute ethanol and distilled water was increased to 4 times, and then white powder samples were obtained under the same drying conditions.

Since both treatment 2 and 3 yielded inclusion complex powders, for the convenience of subsequent UV-visible spectrophotometer detection, the lyophilized 6-G/β-CD inclusion complex powder was dissolved in absolute ethanol. The 6-G in the inclusion complex was completely dissolved by magnetic heating and stirring at 60°C and 800 rpm for 20 minutes. The dissolved solution was filtered through a nylon filter membrane with a pore size of 0.22 μm to remove insoluble β-CD. Subsequently, it was diluted through absolute ethanol to achieve a suitable concentration. The absorbance of 6-G at the maximum absorption wavelength in the above solutions was recorded using absolute ethanol as a blank control.

Results: As shown in Fig. S2, treatments 1 and 3 yielded relatively pure 6-G/β-CD inclusion complexes, but they also had their own advantages and limitations. The advantage of treatment 1 is that it is relatively simple, and does not require a complex vacuum filtration step. However, its limitation is that it is a liquid sample, and the quality of the sample is difficult to be accurately recorded, so the relative proportion of 6-G and β-CD cannot be inferred. The advantage of treatments 2 and 3 is the use of freeze-drying to convert the liquid sample to a solid powder, which helps to record the quality of the sample accurately. At the same time, to ensure adequate and as pure as possible sample volume, a vacuum suction filter device is used to leach the samples. While treatment 2 (2 leaches) could not completely remove 6-G that is not encapsulated by β-CD, so treatment 3 (4 leaches) is more appropriate.

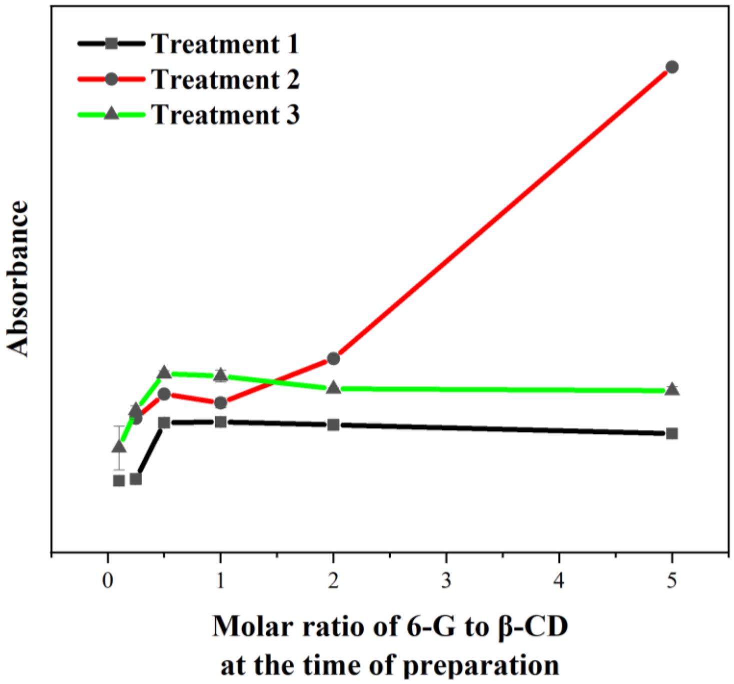

Figure S2. UV absorption intensities of 6-G/β-CD inclusion complexes were obtained by three treatments
